# Solitary bee larvae modify bacterial diversity of pollen provisions in the stem-nesting bee, *Osmia cornifrons* (Megachilidae)

**DOI:** 10.3389/fmicb.2022.1057626

**Published:** 2023-01-09

**Authors:** Jordan G. Kueneman, Jessica Gillung, Maria T. Van Dyke, Rachel F. Fordyce, Bryan N. Danforth

**Affiliations:** ^1^Danforth Lab, Department of Entomology, Cornell University, Ithaca, NY, United States; ^2^Lyman Entomological Museum, McGill University, Sainte-Anne-de-Bellevue, QC, Canada

**Keywords:** bees, brood cell, Megachilidae, development, pollen, microbiome, larvae–development, plant pathogen

## Abstract

Microbes, including diverse bacteria and fungi, play an important role in the health of both solitary and social bees. Among solitary bee species, in which larvae remain in a closed brood cell throughout development, experiments that modified or eliminated the brood cell microbiome through sterilization indicated that microbes contribute substantially to larval nutrition and are in some cases essential for larval development. To better understand how feeding larvae impact the microbial community of their pollen/nectar provisions, we examine the temporal shift in the bacterial community in the presence and absence of actively feeding larvae of the solitary, stem-nesting bee, *Osmia cornifrons* (Megachilidae). Our results indicate that the *O*. *cornifrons* brood cell bacterial community is initially diverse. However, larval solitary bees modify the microbial community of their pollen/nectar provisions over time by suppressing or eliminating rare taxa while favoring bacterial endosymbionts of insects and diverse plant pathogens, perhaps through improved conditions or competitive release. We suspect that the proliferation of opportunistic plant pathogens may improve nutrient availability of developing larvae through degradation of pollen. Thus, the health and development of solitary bees may be interconnected with pollen bacterial diversity and perhaps with the propagation of plant pathogens.

## Introduction

Both solitary and social bees have been shown to host diverse communities of microbial taxa both in their guts, as well as in their pollen/nectar provisions (Gilliam et al., 1989; Mattila et al., 2012; Anderson et al., 2014; Kwong and Moran, 2016; Dharampal et al., 2020). While the adult gut microbiome may play an important role in adult fitness (Koch and Schmid-Hempel, 2011; Kwong and Moran, 2015; Raymann et al., 2017; Rutkowski et al., 2021), it is the bacterial community of the pollen/nectar provisions that plays a key role in larval growth and development (Dharampal et al., 2020). Mounting evidence across diverse bee species suggests that the pollen/nectar provisions in both solitary and social bees host diverse bacterial and fungal taxa (Gilliam, 1979a,b, 1997; Gilliam et al., 1990; Rosa et al., 2003; Pimentel et al., 2005; Vannette et al., 2013; McFrederick et al., 2014) and that these microbes are vital to larval development (Vannette et al., 2013; Steffan et al., 2019; Dharampal et al., 2020), immune function (Mattila et al., 2012; Kaltenpoth and Engl, 2014; McFrederick et al., 2014), resistance to disease (Raymann and Moran, 2018), and overall fitness (Steffan et al., 2017; Dharampal et al., 2019, 2020; Voulgari-Kokota et al., 2019a,b; Cohen et al., 2020; Rothman et al., 2020).

The bacterial diversity from brood cell provisions of species of Megachilidae are particularly well-studied because megachilids construct above-ground, stem- and cavity-nests that can be easily sampled. For example, Mohr and Tebbe (2006) documented the bacterial community in pollen provisions of *Osmia bicornis* and found several bacterial genera including *Sphingomonas*, *Ralstonia*, *Burkholderia*, and *Acinetobacter*. Studies on the nest chambers of *Osmia bicornis* found bacterial families Burkholderiales, Clostridiaceae, Enterobacteriaceae, and Acetobacteraceae, and bacterial genera *Bacillus* and *Paenibacillus* (Keller et al., 2013; Voulgari-Kokota et al., 2019c), whereas, a study of cultured bacteria from nest contents of *Osmia cornuta*, revealed seven prevalent bacterial genera: *Bacillus*, *Lactobacillus*, *Paenibacillus*, *Clostridium*, *Serratia*, *Pantoea*, and *Curtobacterium* (Lozo et al., 2015). Additionally, a comparative study of larvae and pollen provisions from three genera of Megachilidae found a monophyletic *Lactobacillus* clade shared by this group (McFrederick et al., 2017). Thus, there is both overlap and variable bacterial diversity across the pollen provisions of related megachilid species. These bacterial taxa are largely considered to be of environmental origin, obtained primarily through foraging for pollen and nectar on host-plant flowers (Vannette, 2020; Keller et al., 2021). Furthermore, through comparisons of field collected samples, several studies have documented changes in the bacterial community in both the pollen provisions and larvae (Mohr and Tebbe, 2006; Keller et al., 2013; Voulgari-Kokota et al., 2019b), but these studies lacked a standardized experimental design and a direct comparison of pollen provisions with and without larvae. Therefore, the influence of developing bee larvae on the microbial environment of pollen provisions has yet to be fully assessed.

Bees have been shown to depend on microbial symbionts that colonize their gut and on microbes fermenting and metabolizing pollen provisions. Notably, past studies using trophic biomarkers have shown that microbes may be direct prey for bees making bees omnivorous (feeding on both plant and microbial-derived food) rather than strictly herbivorous (Steffan et al., 2019). This appears to be true for solitary bees where microbes have been shown to be an important source of larval nutrition and the microbial activity in pollen provisions helps unlock nutrients, trapped within the rigid, indigestible exine of the pollen (Steffan et al., 2019). Experiments in which microbes are eliminated from the pollen provisions through sterilization indicate that the presence of the naturally occurring microbial community is essential for larval growth and development (Dharampal et al., 2019, 2020).

Given the importance of the brood cell microbiome to larval growth and development, one might expect solitary bee adults and larvae to influence the richness and composition of the brood cell microbiome. However, while adult derived inputs, such as glandular secretions, have been shown to produce select antimicrobial properties in pollen microbiome (Cane, 1983), no previous study has directly examined the impact of larval feeding on the microbial community of pollen provisions. In this study, we set out to explore how solitary bee larvae impact the brood cell microbiome in a common, easily manipulated, solitary, stem-nesting bee, *Osmia cornifrons* (Megachilidae). We conducted an experiment to determine (1) the temporal shift of larval and pollen bacterial communities through larval development and (2) whether actively feeding larvae modify the brood cell bacterial community through feeding.

## Materials and methods

### Preparation for bee experimentation

Long term nesting aggregations of *Osmia cornifrons* in the vicinity of Ithaca, NY were used as a source of brood cells for our experiments. In Spring 2018, local populations of adult *O*. *cornifrons* established nests in wooden nesting shelters housing collections of 70–140 empty cardboard tubes with paper nest inserts purchased from Crown Bees (Woodinville, WA, United States). In June 2018, completed nests were overwintered at ambient conditions. In February 2019, nests were examined *via* x-ray imaging, and nests with parasites or high levels of mortality were excluded from the experiment (Pitts-Singer, 2004). Parasite-free nests were established in the field in March 2019 alongside unused nesting tubes. Following emergence of adult males and females, nesting shelters were surveyed daily for nest completion. Once unused nesting tubes were closed by a female, we brought these tubes into the laboratory for our experiments. Closed nests were collected between May 6 and May 27th from two localities.

### Bee sampling and processing

**Figure 1:**
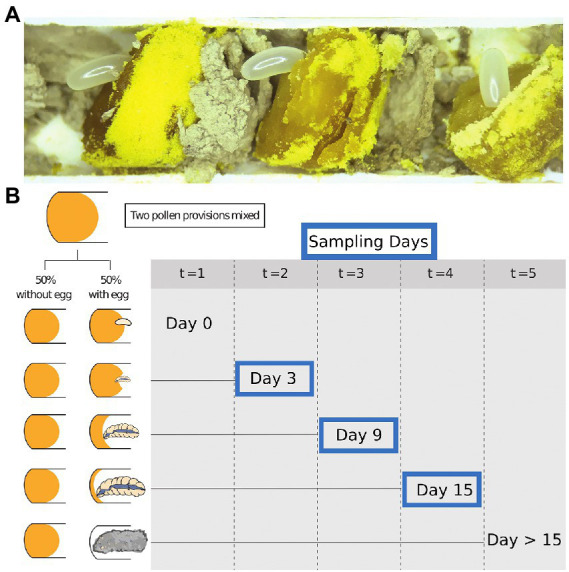
Samples and study design. **(A)** The exposed cells of *Osmia cornifrons*. **(B)** Experimental overview shows sampling time during our experiment: points; (t = 0 = day 0; t = 1 = day 3; t = 2 = day *6*; t = 3 = day 9; t = 4 = day 12; and t = 5 = day 15). Only Day 3, 9, and 15 were selected for bacterial diversity analysis.

We opened recently completed nests of *Osmia cornifrons* containing freshly provisioned pollen and recently laid eggs. Each nest was carefully opened by slicing the paper nest inserts horizontally on each side with a sterile scalpel and removing the top portion to reveal the nest contents. Damaged eggs or larvae were excluded from the experiment. Pollen provisions (approximately 10 per tube; see Figure 1A), were extracted with sterile forceps. Tools were flame sterilized between each sample and working areas were cleaned before and after dissections with 10% bleach.

Undamaged pollen provisions were collected in pairs. Eggs were gently removed and then the two pollen provisions from neighboring cells in the nest were homogenized together using a sterile micro-spatula (Kimura spatula; World Precision Instruments). Pollen balls were combined from brood cells of the same sex, which was determined visually by the mass of the pollen/nectar provision, the size of the brood cell, and the position within the nest. Pollen provisions destined for female offspring are much larger and placed farther from the nest entrance than those destined for males (Figure 1A). The homogenized pollen ball was split in two equal parts and placed into 48-well tissue culture plates (purchased from Falcon). The pollen was gently compacted into the bottom of the well to minimize desiccation and empty wells were used to separate pairs of samples. Subsequently, a male or female egg was returned to one of the pair (pollen from the same sex as the egg), and the other was left without the egg (later described as “pollen with larvae” and “pollen without larvae,” respectively). Plates were stored in an incubator (Percival 500,365) at 27°C. We tracked larval development daily. Pollen and larvae were sampled from the tissue culture plates at 3-day intervals, and we analyze samples for bacterial diversity on day 3, 9, and 15. Most larvae completed development and began spinning cocoons on day 15, so the last sample was taken at the beginning of the spinning larval stage. Samples of pollen and larvae were stored in sterile 2.0 ml screw-cap vials, immediately frozen in liquid nitrogen, and then stored at −80°C for later analysis and amplicon sequencing.

From the pairs of pollen samples described above, we selected representative pairs (no contamination, no parasites, or death) for downstream processing. Sample sizes per group are as follows: day 3, pollen with larvae and pollen without larvae (*N* = 24, 12 pairs) and larvae only (*N* = 12); day 9, pollen with larvae and pollen without larvae (*N* = 23, 11 pairs) and larvae (*N* = 12); day 15, pollen with larvae and pollen without larvae (*N* = 7, and *N* = 11, respectively) and larvae only (*N* = 11; See Figure 1B for experimental design). Total sample numbers by type: pollen with larvae *N* = 30, pollen without larvae *N* = 35, and larvae only  *N* = 35. Sample numbers were slightly reduced at the final observation day, due to the number of individuals that had completely consumed pollen provisions on, or before, sample collection day 15.

### DNA extraction

We extracted DNA from each sample (approximately 100 mg) using Qiagen PowerPlant kits, following manufacturers protocol, including the recommended 10 min of bead beating with the lysis buffer (Galimberti et al., 2014). We homogenized the sample using the Bead Ruptor Elite, set at 1.15 m/s, for 10 min. We found this setting was sufficient for complete mixing and mechanical disruption of the pollen sample and the larvae. An extraction control was added during each extraction event, approximately once every 48 extractions (once per 50 reaction kit), and these extraction controls were included in library preparation and sequencing. Our final elution volume was 60 μl, chosen to increase DNA concentration and improve quantification and down-stream sequencing.

### Sequence processing

To assess the microbiome of samples collected throughout our experiment, 100 pollen and bee larval samples were submitted for 16S amplicon sequencing. Library preparation and sequencing was performed at the UC Davis Department of Medical Microbiology laboratory using the following protocol. Primers 799F (CMGGATTAGATACCCKGG) and 1193R (AGGGTTGCGCTCGTTG) were used to amplify the V5–V7 domain of the 16S rRNA using a two-step PCR procedure. This region was chosen to minimize amplification of plant chloroplasts (Beckers et al., 2016; Thijs et al., 2017). A detailed description of our two step PCR procedure is provided in the Supplementary materials. The final product was quantified on a Qubit instrument using the Qubit High Sensitivity dsDNA kit (Invitrogen), and individual amplicons were pooled in equal concentrations. The pooled library was cleaned utilizing Ampure XP beads (Beckman Coulter) and then checked for quality and proper amplicon size on an Agilent 2100 Bioanalyzer (Agilent Technologies). The library was quantified *via* qPCR followed by 300-bp paired-end sequencing using an Illumina MiSeq instrument (Illumina) in the Genome Center DNA Technologies Core, University of California at Davis, CA, United States.

The amplicon sequence data was exported as Fastq files and were demultiplexed with dbcAmplicons from https://github.com/msettles/dbcAmplicons using miniconda. Then, the input files barcode sheet, primer sheet, and sample metadata were validated. The resulting amplicon sequence data was imported into QIIME2 (v2021.4, Bolyen et al., 2019). We truncated forward reads at 260 bp and reverse reads at 160 bp, based on the length of our fragment and visual inspection of the error profiles and quality scores. We used DADA2 to join reads, de-noise, and dereplicate sequences, including the removal of chimeric sequences, quality filtering, and joining of paired ends (Callahan et al., 2016). Taxonomy was assigned using the vsearch referencing SILVA version silva138 with 99% identity (Bokulich et al., 2018). We extracted reference ASV with “classify-sklearn” and aligned sequences with MAFFT (align-to-tree-mafft-fasttree) generating a rooted phylogenetic tree (Bolyen et al., 2019). Following taxonomy assignments all sequences matching to chloroplast, mitochondria, and any sequences left as unspecified were removed. Bee larval samples experienced moderate host amplification and these sequences were identified in the 16S dataset and filtered from those samples. DNA extraction control samples returned little to no amplification, did not exhibit evidence of contamination and were subsequently excluded from downstream analyses. We filtered out ASVs with <10 sequences per sample.

### Richness, evenness, and composition analyses

Alpha and beta diversity metrics were calculated using QIIME2 (2021.4) and computed using the diversity plugin in QIIME2. To assess differences in alpha diversity and evenness we report and visualize “observed features,” the total number of unique ASVs calculated by sample type. We utilize this metric to capture shifts in richness that consider nearly all microbes found in pollen, including changes in the presence and absence of comparatively rare microbes between and across sample types. The significance of differences for all alpha diversity and evenness metrics were calculated using Kruskal-Wallis tests, followed by pairwise Wilcoxon rank-sum tests with Benjamini-Hochberg FDR (BH) corrections, when significant differences were observed (Kruskal and Wallis, 1952; Benjamini and Hochberg, 1995).

To analyze compositional differential abundance between groups, we investigated the bacterial community structure, using weighted and unweighted UniFrac metrics (Lozupone and Knight, 2005; Lozupone et al., 2007). Significant differences for beta diversity metrics were calculated using a permutational multivariate ANOVA (PERMANOVA) followed by pairwise PERMANOVAs with BH correction when significant differences were observed over more than two factors. To visualize differences in beta diversity metrics, we used principal coordinate analysis (PCoA).

### Proportional abundance tables of pollen without larvae, pollen with larva, and larvae

To visualize and compare the overall taxonomic structure of the bacterial communities in our experiment, we plotted the relative abundance of ASVs matching to the top 10 most abundant bacterial genera by sample type (pollen without larvae, pollen with larvae, and larvae only). We consider the top 10 most abundant bacterial genera by sample type to be the dominant bacterial taxa. To compare proportional abundance by sample type and by experimental time point, taxonomic tables were grouped in QIIME2. While we summarize the data tables at the level of genera, several taxa were not identified to the level of genus and remain described at the order and family level. The dominant bacterial taxa were generally shared across sample types. To visualize the bacterial composition of pollen and larvae across time, we plotted the relative abundance of the combined 12 most dominant bacterial taxa in R.

### Differential abundance testing

To analyze differential abundance of bacterial taxa between pollen samples with and without larvae, we first compare samples from day 9 and day 15. These sampling days independently reveal significant differences in bacterial diversity between sample types (pollen with and without larvae) and combined they offer a more robust analysis of differential features between the two sample types.

We analyze differential features using a compositionally aware method Songbird QIIME2 plugin (Morton et al., 2019). This approach works from our unrarefied dataset and includes all 41 relevant samples: pollen with larva (*N* = 18) and pollen without larvae (*N* = 23). First, the sample data were split into a test set and a training set. Songbird trains a null model and a multinomial model on the training data for each set of metadata explored then predicts and tests this against the test dataset. We quantify the model’s performance, compare the models, and visualize the model’s ability to differentiate between the pollen groups in question. Microbes that significantly contribute to differences between pollen with and without larvae were extracted. Next, we use DESeq2 package in Phyloseq available in R (R Core Team, 2021) following Kapheim et al. (2021). This approach uses a rarified data table and calculates the differential abundance of taxa specified at desired taxonomic levels and significance thresholds. Here, we calculate the maximum log fold change for differential taxa and visualize the data (R Core Team, 2021).

## Results

### Research questions and objectives overview

The goal of our experiment was to document the natural progression of microbial community growth over time in bee brood cells for: pollen with larvae, pollen without larvae, and for larvae only (Figure 1). Our analyses of pollen with larvae, pollen without larva, and larvae only fell into five primary sets of comparisons. To determine the temporal change in bacterial diversity in the absence of larvae we compared (1) pollen only through the experiment. To determine the temporal change in bacterial diversity in the presence of larvae we compared (2) pollen with larvae through the experiment. To determine temporal change in bacterial diversity in larvae we compared (3) larvae only through the experiment. To assess whether actively feeding larvae modify the brood cell bacterial community through time, we compared (4) alpha and beta diversity metrics of bacterial diversity of pollen, both with and without larvae, through the experiment. Finally, we (5) identified bacterial taxa with differential abundance between samples of pollen with and without larvae.

**Figure 2:**
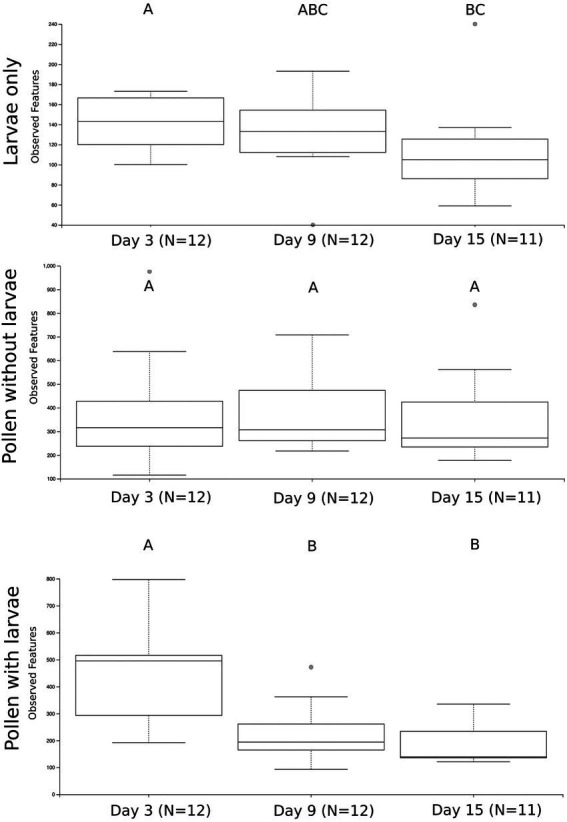
The number of unique bacterial ASVs summarized by the three treatment groups and sampling time points. Significance is denoted by **(A–C)**. Significant differences in bacterial richness across time points were found using a Kruskal Wallis test for larvae only (H = 4.69, *p* = 0.096) and pollen with larvae (H = 11.53, *p* = 0.003), but not for pollen without larva (H = 0.358, *p* = 0.836).

**Figure 3:**
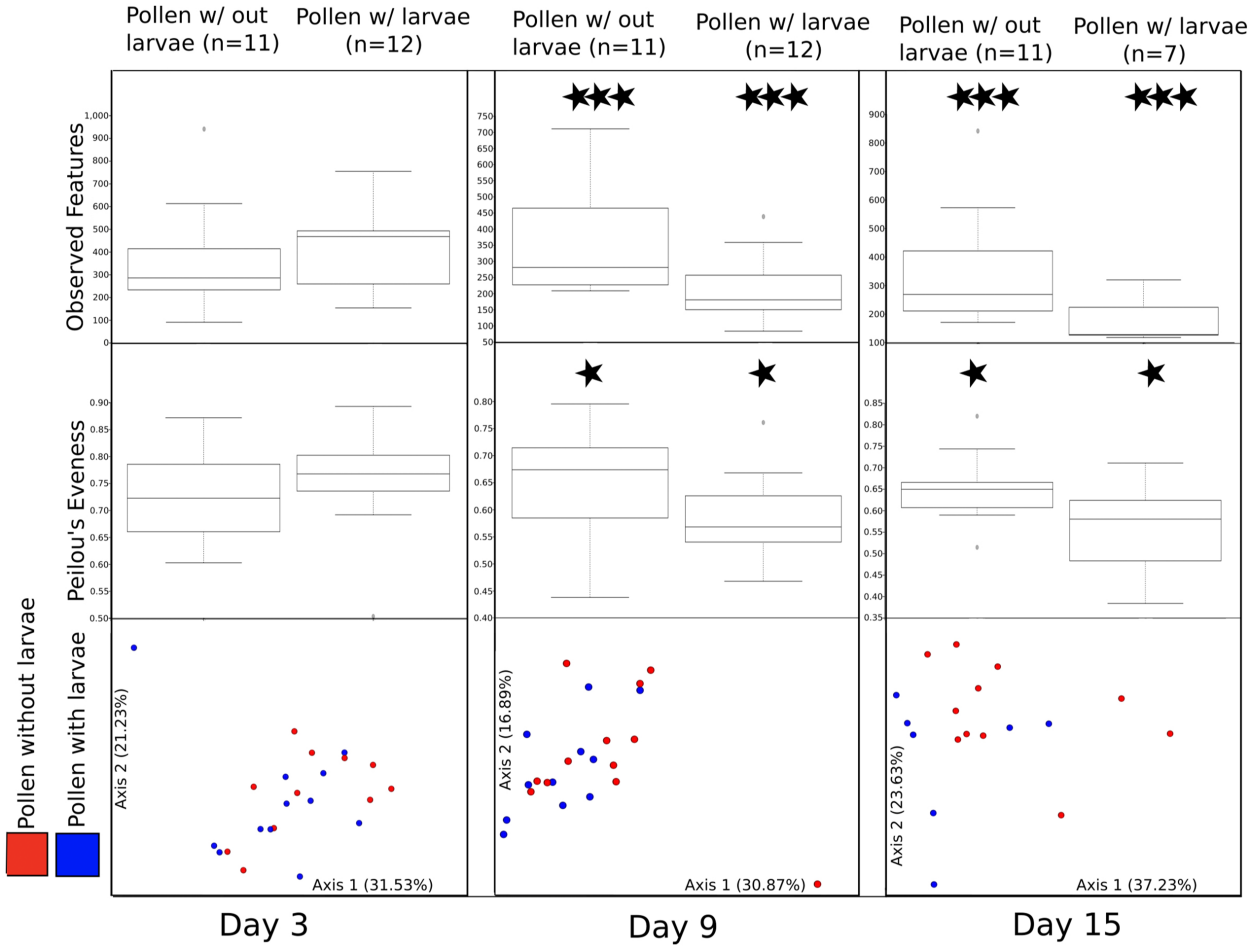
The proportional abundance of dominant bacterial ASVs of pollen and *Osmia cornifrons* summarized across sampling days. Proportional abundances are displayed for three treatment groups, larvae only, pollen with larvae, and pollen without larvae.

**Figure 4:**
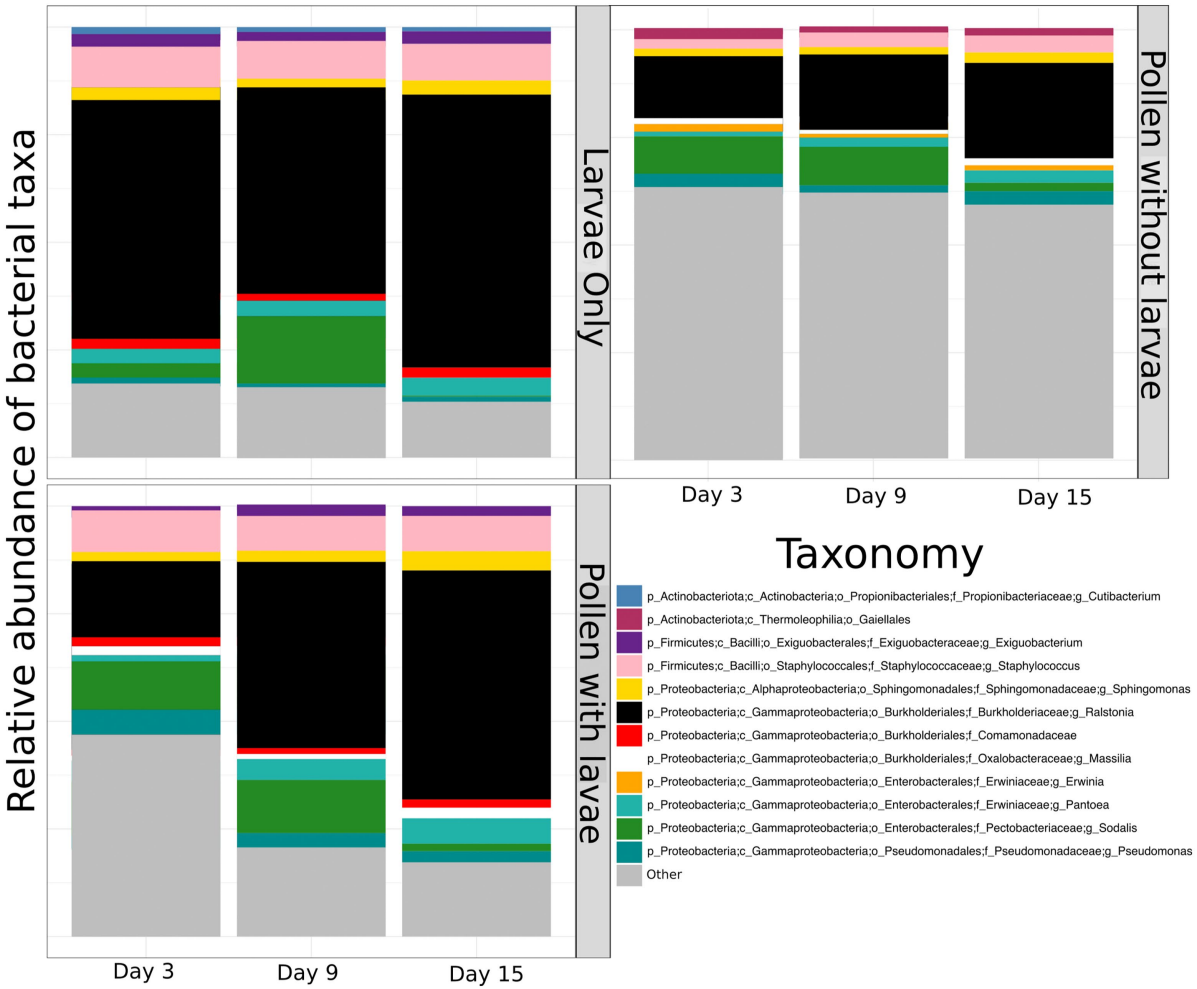
A comparison of bacterial diversity between pairs of pollen masses that were combined and then separated returning one larva to each pair. All figures (observed features, Peilou’s evenness, and beta diversity) are summarized by three experimental time points (day 3, day 9, and day 15). Moderately significant is denoted by one star (*) and significant differences are denoted by three stars (***).

We find that the presence of a bee larva exerts a selective force on the bacterial diversity of pollen throughout bee development (Figures 2–4). After initial filtering, our dataset comprised 99 samples and the resulting 16S ASV table held 4,751,528 sequences, with a median frequency of 38,221 sequences per sample and 13,518 unique bacterial features. After reviewing alpha rarefaction plots, we were able to capture most of the bacterial diversity using a rarefaction depth of 4,690 sequences per sample which only resulted in one sample (pollen without larvae on day 3) dropping below that threshold (Supplementary Figure 1). This rarefaction depth maximizes our exploration of community composition while excluding only a minimal number of samples from our experiment.

### Richness and compositional assessment of brood cell bacterial diversity

We first compare the richness of pollen with larvae, pollen without larvae and larvae only by combining all samples and all time points in our experiment (Supplementary Figure 2A). We calculate the richness of observed ASVs and evaluated them with Kruskal-Wallis and then pairwise Wilcoxon rank-sum tests with Benjamini-Hochberg corrections. At this level of comparison, we see significant differences in the richness of all three sample types (Kruskal Wallis; H = 53.45, *p* = 2.44) and all pairwise comparisons are significant: pollen with larvae compared with pollen without larvae (H = 3.01, *p* = 8.26 e−12), pollen with larvae compared with larvae only (H = 29.97, *p* = 4.38 e-8), and lastly pollen without larvae compared with larvae only (H = 45.51, *p* = 1.52 e−11). We calculate the richness of sample types across three timepoints (day 3, day 9, and day 15) and find that the richness of pollen with larvae and larvae only change significantly throughout larval development, but pollen without larvae is unchanged throughout our experiment (Figure 2). Pollen with larvae was significantly different between day 3 and day 9 (H = 8.02, *p* = 0.0046), and day 3 and day 15 (H = 7.78, *p* = 0.005), but not significantly different between day 9 and day 15 (H = 0.593, *p* = 0.441). The overall difference of pollen with larvae across the three time points was significant (H = 11.53, *p* = 0.003). Pollen without larvae was not significantly different across any of the three time points, and the overall difference across groups was not significant (H = 0.358, *p* = 0.836). Larvae only were significantly differentiated between day 3 and day 15 (H = 4.908, *p* = 0.027), however, overall differences between all three-time points were not significant (H = 4.69, *p* = 0.096).

To compare and visualize the bacterial composition of the bees in our study, we plot the proportional abundance of the 12 most abundant bacterial taxa (referred to as “dominant” bacteria) and grouped all additional taxonomic groups into the category “other” (Figure 3). We found that most dominant taxa identified remain relatively equivalent and consistent across sample types and through time. However, the proportional abundance of *Ralstonia* increases considerably over time in pollen with larvae. Conversely, the proportional abundance of *Sodalis* decreases with time in all groups. One bacterial taxon, identified only to the level of Comamonadacae was considered dominant only in samples with larvae, and another bacterial taxon, *Erwinia*, was considered dominant only in samples without larvae. Most importantly, the proportional abundance of all “other” taxa decreases in time for all groups and this is particularly evident for pollen with larvae (Figure 3). Additionally, we confirm that *Ralstonia*, a dominant member of our pollen microbial community, is a valid member, and we verified that larval feces were not a significant contributor to bacterial shifts that occur in the pollen microbiome during larval development. For brevity, results regarding the diversity of *Ralstonia* and feces in pollen samples are available in the Supplementary results.

### Composition assessment of pollen with and without larvae, and larvae only

To assess the bacterial composition of our sample types and differences in beta diversity between and among groups, we analyzed weighted UniFrac distance matrices and visualized the beta diversity using PCoA. We use weighted UniFrac because it is robust against modest changes in the abundance of individual bacterial taxa. When we compare overall differences in our sample types (pollen with larvae and without larvae, and larvae only), we observed significant differences in sample type (Supplementary Figure 2B; *F* = 10.93, *p* = 0.001). Next, we compared our sample types across three sampling events in our experiment (day 3, day 9, and day 15). We find that pollen with and without larvae, and larvae only, all change across sampling days (Supplementary Figure 3). However, pollen with larvae changed the most (*F* = 5.560, *p* = 0.001) and larvae only changed the least (*F* = 2.42, *p* = 0.012).

### Pollen with and without larvae at each sampling day

To better assess the direct effect of larval development on pollen masses with and without larval development, we paired these samples at each time point (Figure 4; see methods for homogenized pollen masses). First, we compare the bacterial richness (observed features) for these paired samples at each of the three time points (day 3, day 9, and day 15). We find no significant difference at day 3 (H = 0.592), but significant differences between pollen with and without larvae on day 9 (H = 6.06, *p* = 0.0138) and day 15 (H = 4.93, *p* = 0.0264). Second, we compared Pielou evenness between paired samples. We detect no significant difference between the bacterial evenness on day 3 (H = 0.592, *p* = 0.442), and only a moderately significant reduction in evenness of pollen with, compared to pollen without larvae, for time point day 9 and day 15 (H = 2.97 *p* = 0.085: and H = 2.81, *p* = 0.094), respectively. Third, we compared the bacterial composition (beta diversity) using weighted UniFrac, and we detect no significant difference between pairs of samples using PERMANOVA at day 3, or at day 9 (*F* = 0.86, *p* = 0.5 and *F* = 1.40, *p* = 0.1, respectively). However, marginally significant differences were detected between sample pairs at day 15 (*F* = 2.03, *p* = 0.06). A similar pattern is observed for unweighted UniFrac, which considers all taxa equally, regardless of their abundance. Again, we detected no significant difference between pairs of samples using PERMANOVA at day 3 (*F* = 0.79, *p* = 0.82). However, significant differences were detected between pairs of samples at day 9 and day 15 (*F* = 1.52, *p* = 0.011 and *F* = 1.70, *p* = 0.012, respectively). Differences between pollen without larvae and pollen with larvae are even more evident when samples collected on days 9 and 15 are combined (Supplementary Figure 4).

### Taxonomic differences between pollen with and without larvae

We employ two approaches to explore taxa that significantly and non-significantly differentiate samples of pollen with and without larvae. Because differences between these groups are detected at day 9 and consistent on day 15, we combine these days to increase our ability to detect bacterial taxa with differential abundance between these two-sample types. A detailed justification is provided in the Supplementary materials. In our first approach, we extracted the taxa from the numerator of the balance table generated using Qurro plots (Quantitative Rank/Ratio Observations) that integrate the Songbird model differentials that predicted samples of pollen with and without larvae. The predictive model explains 22 percent more variation than the null model. The differential table produced with Qurro highlights 27 taxa that contribute to the predictive power of the multinomial model. The bacterial taxa identified are summarized in Supplementary Figure 5. Using a second approach, DESeq2 in phyloseq, we again identified the taxa that differed between samples of pollen with and without larvae. This approach assigns a direction to the features differential abundance, and we present this data with two figures: (Supplementary Figure 6A) shows taxa that are significantly different (*p* = 0.05) between the two groups and (Supplementary Figure 6B) shows the general pattern (*p* = 0.5) observed across diverse bacterial families. While there is significant overlap in the taxa that contribute most to differences between the groups identified using Qurro and DESeq2 (e.g., genera *Gaiella*, *Massilia*, *Pseudomonas*, and *Bradyrhizobium*, as well as unidentified; 17–14, and MND1), each approach also reveals additional bacterial taxa that separately contribute to differences in the sample types. Importantly, the results of DESeq2 suggest *Ralstonia* had only a modest increase in abundance, and the results of Qurro confirm that *Ralstonia* contributes little to differences between the sample types. Thus, *Ralstonia* is more of a constant than it appears and observed differences in its proportional abundance are, in part, the result of decreases in other taxa.

## Discussion

### General overview

Solitary bees, and their associated mutualistic and beneficial microbes, support plant diversity and the health of diverse ecosystems (Frison et al., 2011; Vanbergen and Initiative, 2013). Understanding how microbes interact with developing bee larvae, and vice versa, is essential for understanding how microbes impact bee health (Engel et al., 2016). General principles regarding interactions between pollen and larval microbes through bee development are limited yet needed to better understand the biology of solitary bees. To the best of our knowledge, no previous studies have evaluated the bacterial diversity of pollen provisions through time while controlling for the presence of larvae. Furthermore, no previous study has described the bacterial diversity of pollen provisions or the larval microbiome of *Osmia cornifrons*, an agriculturally important pollinator.

Our study utilized a controlled experimental design to assess the impact of larval development on pollen microbes and that of pollen microbes on larval gut microbes. By characterizing the bacterial diversity of pollen provisions with and without larvae, as well as the bacteria within larvae, sampling repeatedly throughout larval bee development, we obtained important insights into the microbial ecology within the closed “mini ecosystem” of the bee brood cell (Biani et al., 2009). Our study, like all other published work on *Osmia* microiomes, did not amplify fungal and micro eukaryotic members of the brood cell. However, only a limited scope of published work, pertaining to a specialized social stingless bee, found fungi to be a critical component to larval nutrition (Menezes et al., 2015). Rather we contend, apart from several fungal pathogens, bacterial diversity of the pollen provision is most relevant to *Osmia* developmental biology. Here, we found abundant bacterial diversity in pollen provisions of *Osmia cornifrons*, higher than what has previously been reported for bees in the family Megachilidae, and we found that the bacterial diversity in larvae is a reduced subset of what is available in the pollen provision. Additionally, we found evidence suggesting that developing larvae exert a selective pressure on the pollen microbiome through time—larval feeding appears to diminish the rare bacterial taxa in the pollen community. Furthermore, we discovered many bacterial taxa in the pollen provisions of *O*. *cornifrons* that correspond to known plant pathogens, suggesting that the bee brood cell provisions may serve as a repository for plant pathogens (Rothman et al., 2019; Kapheim et al., 2021).

### A comparison of the bacterial diversity across *Osmia* species

The bacterial diversity within pollen provisions of *Osmia corniforns* partially matches what is known about the bacterial diversity of other *Osmia* species, as well as bees within the family Megachilidae more broadly. For example, we find higher bacterial richness in the provisions of *O*. *corniforns* compared with several other *Osmia* species (Keller et al., 2013; McFrederick et al., 2014; Lozo et al., 2015; Voulgari-Kokota et al., 2019a,b), yet, similar to richness found in *O*. *lignaria and O*. *ribifloris* (Rothman et al., 2020). Like our central finding, that larvae reduce bacterial diversity of pollen provisions, decreasing bacterial diversity in the pollen provisions of *O*. *caerulescens* through larval development was reported (Voulgari-Kokota et al., 2019a) Additionally, bacterial structure across sample types was paralleled in our results, such that some proportionally abundant bacteria were present in pollen (e.g., *Erwinia*), but not in larvae (Voulgari-Kokota et al., 2019b). Indeed, bacterial taxonomy of *O*. *corniforns* pollen provisions was generally congruent with the aforementioned studies of Osmia species. Specifically, bacterial phylum Actinobacteria, Firmicutes, and Proteobacteria were considered dominant in *O*. *cornifrons* and abundant in other *Osmia*, and at higher resolution, bacterial orders Burkholderiales, Enterobacteriales, Clostridiales, and Pseudomonadales, as well as bacterial genera *Pantoea*, *Sodalis*, and *Massilia* were also shared (Keller et al., 2013; Lozo et al., 2015; Rothman et al., 2019, 2020; Voulgari-Kokota et al., 2019b; Cohen et al., 2020). Further, *Paenibacillus*, a bacterial pathogen of honey bees (Ebeling et al., 2016) and a potential pathogen of *O*. *bicornis* (Keller et al., 2013, 2021) was also consistently found in low abundance across samples of *O*. *cornifrons* pollen and larvae.

### Bacterial taxa and diversity patterns of *Osmia* not found in *Osmia cornifrons*

Despite general similarities in bacterial diversity patterns and bacterial taxa found across species of *Osmia*, there exist notable differences in the results from this study compared to previous, primarily field-based, studies. For example, one study reported increasing bacterial diversity in the larvae of *O*. *caerulescens* and the pollen and larvae of *O*. *bicornis through time* (Voulgari-Kokota et al., 2019b). This is dissimilar to our experimental results, which showed decreasing bacterial diversity through time, and thus inconsistent with our view of a closed mini-ecosystem, and our findings that microbial diversity is lost in the presence of a larva.

In our study, more bacterial orders contribute to the total bacterial diversity of pollen provisions, compared with other studies. Nevertheless, several bacterial groups were underrepresented compared with other studies of *Osmia* (Keller et al., 2013; Lozo et al., 2015; McFrederick et al., 2017; Rothman et al., 2019; Vuong and McFrederick, 2019). Specifically, *Acinetobacter* (a common flower bacteria), reported in *Osmia* (Keller et al., 2013; Cohen et al., 2020), was generally absent in our experimental samples. Such differences could arise from biological differences of species of bees and their microbes as well as in their pollen provisions. Similarly, *Lactobacillus* a common microbial member in bee nest environments and adult bees was minimal in *O*. *cornifrons* when compared with microbial studies of other *Osmia* and other Megachilidae (McFrederick et al., 2017; Vuong and McFrederick, 2019; Voulgari-Kokota et al., 2019c). The lack of a dominant *Lactobacillus* and the more specifically *Apilactobacillus* is curious, and perhaps calls into question the generality of this bee-microbe association across diverse groups of solitary bees. Likewise, bacterial genera *Bartonella* and *Bacillus* which include both symbionts and generalist pathogens (Bulla et al., 1975; Gilliam, 1997; Segers et al., 2017), found frequently in both social and solitary bees (Engel et al., 2012; Keller et al., 2013; Lozo et al., 2015), were absent in *O*. *cornifrons*.

Conversely, many bacterial taxa found in our experimental analysis of *O*. *cornifrons* are absent from published studies of *Osmia* or related Megachilidae. However, there is limited utility in reporting all bacterial taxa not identified elsewhere, and rather, we emphasize that the diversity of *O*. *cornifrons* is robust, and potential plant pathogens make up a substantial proportion of the bacterial composition in pollen provisions. This is particularly evident in the presence of a feeding larva, a result not highlighted elsewhere. While we have yet to determine the consequences of these microbes on larval development or the transmission of these taxa back to plants in the spring, we provide a synthesis of the relevant taxa below.

### Bacterial diversity assessment of *Osmia cornifrons* brood cells

We compared both the richness and the composition of the bacterial community for pollen with larvae, pollen without larvae and of larvae only (Supplementary Figures 2A,B). We found significant differences in the richness and composition across these groups of samples and all pairwise comparisons. These results suggest that larvae feeding on pollen significantly reduce the richness of bacteria in the pollen provisions. The nearly 2-fold reduction in bacterial richness between pollen with and without larvae, compared to the richness found in the larval samples themselves, suggests that larvae are not taking up and maintaining pollen bacteria in their gut indiscriminately. Rather, only a portion of the total bacterial diversity is detected in their gut. While it is possible that *O*. *cornifrons* gains little from the microbial environment of the brood cell, existing evidence from a congeneric species suggests that most bacterial taxa are consumed and microbe-derived amino acids and lipids are detectible at high levels in the musculature and fat body of adult bees (Dharampal et al., 2020). Due to the way larvae feed, consuming nearly all pollen in the stored pollen provisions, it is unlikely that they are selectively feeding on certain microbial taxa. Rather, we hypothesize that (1) rare taxa are lost during metabolism, (2) that their gut is selective against nearly all microbes and only the dominant bacterial taxa are recovered, and/or, (3) the chemistry of the larval gut selects against certain bacterial taxa, favoring others. When we compared the species richness of pollen with and without larvae, and larvae only through our experiment we also see evidence that larvae exert a selective force on the pollen microbiome. This may occur due to selection against microbes caused by larvae, perhaps through secretions, or because some bacterial taxa are able to replicate in this mini-ecosystem and other are not. Reduced pollen mass through feeding may also haphazardly remove rare microbes, but this is likely insufficient to fully explain the decrease in pollen bacterial diversity, as sufficient pollen material was recovered at all time points, and we presume the distribution of bacterial diversity in the pollen mass is generally homogeneous. Taken together, we found that the bacterial richness of larvae only and pollen with larvae are significantly reduced through time. This was not the case with pollen provisions incubated without larvae, which remained unchanged over time.

### Pollen with and without larvae at each time point

To better understand the influence of developing larvae on the microbiome of pollen provisions, we utilized direct comparisons of pollen with and without larvae through time. We found a significant reduction in the bacterial richness of pollen with larvae, compared to pollen without larvae by day 9 and, while still declining through day 15, the difference between day 9 and 15 was non-significant. This suggests that the larval effect on the bacterial diversity of pollen is minimal at first but is substantial by day 9. It also suggests that the reduction in pollen bacterial diversity, driven by larval development is bounded, and that at least 1/3rd of the bacterial diversity is resilient and can withstand the impact of larval feeding. Similarly, we found a moderately significant reduction in the evenness of the bacterial community of pollen with feeding larvae, compared to pollen without larvae by day 9. A decline in evenness implies that some bacterial taxa are becoming increasingly common, and we observe that the diverse rare taxa are declining or disappearing; a result supported by our comparisons of bacterial richness in pollen provisions with and without larvae. We find that bacterial composition (weighted UniFrac) shows only a marginally significant difference between pollen with and without larvae by day 15. Taken together the bacterial composition of pollen with larvae appears to be increasingly differentiating from that of pollen without larvae over time, but the largest shifts occur around the mid-point of larval development.

### Taxonomic differences between pollen with and without larvae

We combined samples of pollen with and without larvae by day 9 and 15 to increase our ability to detect differences in the bacterial community composition in the presence and absence of feeding larvae. Utilizing this more robust approach, we detected 16 taxa that are significantly differentiated (Supplementary Figure 6A), and only three taxa that were enhanced. Thus, the presence of larvae does not enhance very many microbes, in fact only two taxa strongly benefit from larval presence. One of these, the genus *Cutibacterium* (known anaerobic bacteria), likely colonizes the larvae itself, and the other, the genus *Massilia* (an aerobic bacteria previously found to be plant associated Ofek et al., 2012), may increase as the result of reduced competition within the pollen microbiome. The differential abundance of microbial species reflects the central finding of our experiment—larvae are selecting against diverse groups of bacteria, many of which are rare to begin with. This would explain why individual taxa are not significantly differentiated, but the sum of these reduced taxa is significantly driving the bacterial diversity between these two sample types. To further illustrate this effect, we plotted the families of bacteria that are nonsignificantly (*p* = 0.5) differentiated between pollen with and without larvae (Supplementary Figure 5). Indeed, most bacterial families are reduced in the presence of a larva, while comparatively few are increasing.

### Composition assessment of pollen with and without larvae, and larvae only

We examined the taxonomic composition of bacteria in pollen provisions with and without larvae and larvae only to compare the dominant 12 bacterial taxa found across all sample types. Here, we observed similar taxonomic structure of pollen with larvae and larvae only, and we did not observe several dominant taxa shared by these two groups in pollen without larvae. Specifically, we do not find bacteria in the genera *Exiguobacterium* and *Comamonadaceae* to be dominant in pollen alone. These results suggest that the influence of larval feeding is observable both inside and outside the larva. In larvae only, we observe little to no change in the taxonomic composition of bacteria across sampling days, except for a modest reduction in the proportional abundance in the genus *Sodalis*, which is a common insect endosymbiont (see below; Dale et al., 2001; Chari et al., 2015). Additionally, bacteria in the genus *Cutibacterium* are only found in the larvae, and the isolated nature of *Cutibacterium* suggests that this bacterium is utilizing the host itself. Conversely, bacteria in the genus *Massilia* are only found in the pollen and do not appear to persist in the larvae.

In pollen without larvae, we again saw a relatively modest difference in composition across sampling days and a slight reduction in the proportional abundance of *Sodalis*. This reduction in the proportional abundance of *Sodalis*, as well as its presence in all sample types, suggests that it was specifically introduced by the adult mother at the time of pollen provisioning. We detected 20 unique sequences of *Sodalis*. When we blast the most abundant sequences, we detected sequences closely related to several endosymbionts of insects including an endosymbiont of a chestnut weevil (*Curculio sikkimensis*; Higaki, 2005), an endosymbiont of a parasitic wasp (*Spalangia cameroni*; Betelman et al., 2017), an endosymbiont of a neotropical mealybug (*Puto barberi*; Szklarzewicz et al., 2018), and endosymbionts of stinkbugs (*Nezara antennata* and *Piezodorus hybneri*; Hosokawa et al., 2015, 2016). The specificity of *Sodalis* in solitary bees is currently unknown, and future work may uncover strains of *Sodalis* to be bee specific or even bee species specific. Furthermore, the near complete absence of *Wolbachia*, a common insect endosymbiont, may suggest potential within-host competition occurs between the two genera.

One notable difference in pollen without larvae is the constant presence of *Erwinia* (a common plant associate and pathogen). *Erwinia* is a genus within the bacterial family Enterobacteriaceae and is generally the sole genus within the family found to be commonly associated with Megachilidae (Voulgari-Kokota et al., 2019b). This group of bacteria may be suppressed by the presence of the larvae, which may aid in preserving the pollen provision. Alternatively, or perhaps in concert with the timing of developing larvae, *Erwinia* may be degrading pollen, thus providing additional or accelerated nutritional value to pollen, a process has been documented for several pollen and flower associated bacteria (Christensen et al., 2021).

By contrast, in pollen with larvae, we see a dramatic increase in the proportional abundance of bacteria in the genera *Ralstonia* and *Pantoea*, and a decrease in *Sodalis*. Most other dominant taxa in pollen with larvae remain relatively stable. The proportional increase in *Ralstonia*, and, to a lesser degree *Pantoea*, appears to result from the significant decrease in non-dominant bacterial taxa, especially in the presence of larvae.

### Plant pathogens in the pollen microbiome

Our analysis of the pollen microbiome of *Osmia cornifrons* revealed the presence of bacterial sequences that match closely to diverse plant pathogens. These presumed pathogens make up a substantial portion of the total bacterial sequences in pollen provisions, and are much higher in their proportional abundance when compared with related bee species (Keller et al., 2013; McFrederick et al., 2014; Lozo et al., 2015; Voulgari-Kokota et al., 2019a,b; Dharampal et al., 2020). In our study, we identify 17 unique sequences matching to bacteria in the genus *Erwinia*, 443 matching to the genus *Pantoea*, 68 matching to the genus *Ralstonia*, and 240 matching to the genera *Pseudomonas*. To improve our understanding of pollen-associated bacterial sequences that match plant pathogenic bacterial genera, we blasted the dominant representative sequences from *Erwinia*, *Pantoea*, *Pseudomonas*, and *Ralstonia*. In doing so, we uncovered sequence matches to previously studied isolates of plant pathogens. We provide a summary for the top five most abundant sequences of each genus known to contain plant pathogens in Supplementary Table 1.

Several of the potential pathogens identified in our study have been reported in other studies of *Osmia* and related bees. Most notably, *Pantoea agglomerans* (a pathogen of pome fruit including apples, pears, nashi, and quince), and a causal agent in fire blight was a dominant species in *O*. *cornuta* pollen provisions (Lozo et al., 2015). Additionally, *Pantoea* more generally was also detected in previous studies of *O*. *bicornis*, *O*. *lignaria*, *O*. *ribifloris*, and *Megachile rotundata* (Keller et al., 2013; Rothman et al., 2019, 2020). *Erwinia*, was also found separately in association with *Osmia lignaria* (Cohen et al., 2020), as well as a small carpenter bee, *Ceratina calcarata* (McFrederick and Rehan, 2016; Dew et al., 2020). *Ralstonia* was present in *O*. *lignaria*, *O*. *ribifloris* (Rothman et al., 2020), and *Osmia bicornis* (Mohr and Tebbe, 2006) as well as *Megachile rotundata* (Rothman et al., 2019). *Pseudomonas* was present in *Osmia bicornis* (Keller et al., 2013) and in *Megachile* and *Osmia* (McFrederick et al., 2017). Since *Osmia cornifrons* is native to Japan, novel pathogenic bacterial associations are likely to exist, and may have been co-introduced into the new range of *Osmia cornifrons* (Hedtke et al., 2015). Indeed, *O*. *cornifrons* has already been implicated in the introduction of *Ascosphaera naganensis* a fungal pathogen of bees that may contribute to declines in related native species (Hedtke et al., 2015; LeCroy et al., 2020).

The discovery of diverse putative plant pathogens accumulating in pollen provisions is intriguing, as the ability of solitary bees to transmit pathogens of plants in orchards and in natural environments represents a substantial knowledge gap. Additionally, how plant pathogens may function in both detrimental or beneficial ways for bees within pollen provisions, and how bees may act in both detrimental and beneficial ways for pathogen transmission dynamics in the spring, are understudied areas. While we currently lack data to test these interactions, we offer several hypotheses. First, plant pathogens may help facilitate pollen degradation that could improve nutritional quality of pollen for feeding larvae. Indeed, experimental evidence of bacteria acting like an external rumen, pre-digesting and enhancing the nutritional quality of pollen for *O*. *ribifloris* was laid out in Steffan et al. (2019) and Dharampal et al. (2019, 2020), and bacterial induced germination of pollen was further detailed by Christensen et al. (2021). It is, therefore, reasonable to assume that a similar mechanism may be at work in *O*. *cornifrons*, and probable that these plant pathogens could possess enzymes or metabolites that may help liberate nutrients from pollen grains. Second, acquisition of potential plant pathogens by *O*. *cornifrons* may simultaneously serve as a sink and/or a source for plant pathogen transmission. By collecting plant pathogens and storing them within brood cells, *O*. *cornifrons* may alter transmission dynamics among plants—a hypothesis also explored in *Megachile rotundata* (Rothman et al., 2019). If these microbes are sequestered, consumed, or do not survive or replicate, pathogen burden of plants may be reduced. If however, *O*. *cornifrons* emerges from their brood cells and carries spores or live cells (perhaps by climbing through infected brood cells) in contact with plants, they may be in part responsible for re-establishing transmission dynamics in the spring. It is conceivable that both processes may be taking place and should be considered when assessing the microbial diversity of pollen provisions in solitary bees. A discussion of horizontal transmission of microbes that occur at flowers and subsequent microbial filtering that may influence *Osmia* pollen bacterial diversity can be found in the Supplementary materials.

### Conclusion

Solitary bees are important pollinators of agricultural crops and diverse flowering plants in natural landscapes. Unlike many floral visitors (flies, moths, and beetles), solitary bees collect and store pollen and nectar as food for their developing offspring. Our experimental design sought to uncover the direct effects of larval feeding on the pollen bacterial community of one solitary bee species, *Osmia cornifrons* that may be representative of bee species in the larger bee family Megachilidae. We find that the bacterial community of developing larvae are relatively stable over the course of larval development, and that the larval microbiome consists of a subset of the dominant bacterial taxa found initially in the pollen provisions. Our results confirm that contact with developing larvae results in a dramatic decrease in bacterial richness of the pollen provision, a decrease in microbial evenness of the pollen provision, and shift in the bacterial composition. These changes in bacterial diversity through larval development are likely the result of selection against the rare bacteria in the system. Indeed, there appear to be very few microbes that benefit from close association with a developing larva and many bacterial taxa are lost along the way. Thus, it does not appear that larval *Osmia cornifrons* utilize specific bacteria internally to support their development, however larvae may benefit from bacteria as a source of nutrition, either directly or indirectly through degradation of pollen. Lastly, bee-microbial interactions likely confer substantial implications for plant pathogen propagation, and still unknown are the feedback mechanisms and reciprocal consequences of plant pathogen propagation for bee health, development and ultimately bee conservation.

## Data availability statement

The datasets presented in this study can be found in online repositories. The names of the repository/repositories and accession number(s) can be found below: NCBI BioProject: PRJNA893014.

## Author contributions

All authors listed have made a substantial, direct, and intellectual contribution to the work and approved it for publication.

## Funding

This project was supported by USDA NIFA (2018-08601) and NSF (NSF-DEB 1929499).

## Conflict of interest

The authors declare that the research was conducted in the absence of any commercial or financial relationships that could be construed as a potential conflict of interest.

## Publisher’s note

All claims expressed in this article are solely those of the authors and do not necessarily represent those of their affiliated organizations, or those of the publisher, the editors and the reviewers. Any product that may be evaluated in this article, or claim that may be made by its manufacturer, is not guaranteed or endorsed by the publisher.
