# Supplementary material for: Solitary bee larvae modify bacterial diversity of pollen provisions in the stem-nesting bee, Osmia cornifrons (Megachilidae)
Source: Front Microbiol. 2023 Jan 9;13:1057626. doi: 10.3389/fmicb.2022.1057626 (PMC9868615; doi:10.3389/fmicb.2022.1057626)
Supplement: Supplementary file 1 [file Data_Sheet_1.docx]

**Supplemental Materials:**

**Supplemental Methods**

*Two step PCR conditions*

In step one of the amplification procedures, both forward and reverse primers contained an Illumina tag sequence and a variable length spacer. To increase diversity and improve the quality of the sequencing run, a linker sequence (TC), and the 16S target sequence, AACMGGATTAGATACCCKG for 799 and ACGTCATCCCCACCTTCC for 1193 were included. Each 25 ml PCR reaction contained 1 Unit Kapa2G Robust Hot Start Polymerase (Kapa Biosystems), 1.5 mM MgCl_2_, 0.2 mM final concentration dNTP mix, 0.2 mM final concentration of each primer and 1ul of DNA for each sample. PCR conditions were an initial incubation at 95°C for 3 min, followed by 28 cycles of 95°C for 45 s, 50°C for 30 s, 72°C for 30 s and a final extension of 72°C for 3 min.

In step two, each sample was barcoded with a unique forward and reverse barcode combination using forward primers (**AATGATACGGCGACCACCGAGATCTACAC**NNNNNNNNTCGTCGGCAGCGTC) with an Illumina P5 adapter sequence (bold), a unique 8 nt barcode (N), a partial matching sequence of the forward adapter used in step one (underlined), and reverse primers (**CAAGCAGAAGACGGCATACGAGAT**NNNNNNNNGTCTCGTGGGCTCGG) with an Illumina P7 adapter sequence (bold), unique 8 nt barcode (N), and a partial matching sequence of the reverse adapter used in step one (underlined). The PCR reaction in step two contained 1 Unit Kapa2G Robust Hot Start Polymerase (Kapa Biosystems), 1.5 mM MgCl_2_, 0.2 mM final concentration dNTP mix, 0.2 mM final concentration of each uniquely barcoded primer and 1ul of the product from the PCR reaction in step one diluted at a 10:1 ratio in water. PCR conditions included an initial incubation at 95°C for 3 min, followed by 9 cycles of 95°C for 30 s, 58°C for 30 s, 72°C for 30 s and a final extension of 72°C for 3 min.

**Supplemental Results and Discussion:**

*Justification for merging day 9 and day 15*

All diversity metrics confirm that pollen with larvae is statistically indistinguishable from pollen without larvae at day 3. Nearly all diversity metrics also find significant or near significant differences between the two sample types on day 9 and on day 15. The consistent finding of reduced richness and reduced evenness that occurs at day 9 and remains stable at day 15, including the significant differences in composition for the two sample types (unweighted unifrac) found at day 9 and 15, suggest that combining these days is appropriate for additional analysis. When we do so, we again find a highly significant difference in compositions (weighted unifrac) of pollen with and without larvae (Supplemental Figure 3). We use this combined comparison to increase our ability to detect bacterial taxa with differential abundance between these two-sample types.

*Verification of microbial taxa Ralstonia*

We conducted a thorough investigation of our most dominant microbial genera, comprised of 70 unique ASVs matching to the genus *Ralstonia.* We conclude that this previously identified plant pathogen (Salanoubat *et al.*, 2002; Álvarez *et al.*, 2010; Wicker *et al.*, 2012), and sometimes identified as a laboratory contaminat (Salter *et al.*, 2014), is indeed a valid member of *Osmia* pollen-associated microbiome. We first, assessed sequences produced by the sequencing center for the previous 900 samples that were generated from numerous sample types including sequences from low microbial biomass samples. No *Ralsonia* was previously detected. Next, we assessed our own low biomass test samples which came from over-wintering *Osmia* adults (known to have little to no bacteria, as well as honeybee samples) and we again find no amplification of sequences matching to *Ralstonia*. Furthermore, when, we reviewed our DNA negative extraction controls, and we see very low total amplification, and even less sequences matching to only one ASV of *Ralstonia*. Most convincingly, compared to our experimental samples, we found 70 ASVs matching to *Ralstonia* and the top three of these taxa yielded 2,074,465 sequences. Taken together our detection of *Ralstonia* in pollen samples of *Osmia* bees more than convinces us that this signal is real and that *Ralstonia* is a dominant member of our pollen bacterial community.

*Larval feces and their contribution to pollen microbial diversity*

Next, we compare microbial diversity of defecated pollen samples compared with non-defecated pollen samples from the 3-day old samples. We find no significant difference in richness: Observed features, H = 2.66, p = 0.102; Shannon, H = 1.5, p = 0.221; Faiths phylogenetic diversity, H = 0.666, p = 0.412. Additionally, we find no difference in composition (weighted UniFrac, PERMANOVA) of defecated pollen samples compared with non-defecated pollen samples from the 3-day old samples (F = 1.06, p = 0.422). As we are unable to replicate this comparison at later sampling days due to ubiquitous defecation, we posit that fecal material, alone, does not significantly impact the microbiome or obscure microbial diversity that otherwise would be detected.

*How diversity patterns of Osmia inform horizonal transmission of microbes*

The generalizable microbial associations across related species of *Osmia*, points to predictable microbial conditions that occurs at floral hubs, and transmission dynamics between microbes and adult bees have been discussed (McFrederick *et al.*, 2017; Adler *et al.*, 2021; Keller *et al.*, 2021; Vannette *et al.*, 2021). While flower associated microbiomes serve as a major source of microbial inoculum, this study provides increasing evidence for a bacterial transmission filter that exists between pollen provisions and larvae. Indeed, larval microbiomes are a subset of their respective pollen microbiota, also found in Voulgari-Kokota *et al.*, (2019) and Kapheim *et al.*, (2021).

This microbial filtering that begins when adult bees visit flowers, continues through bee development, and determines microbial niches that have strong selective consequences for solitary bees (Keller *et al.*, 2021). Indeed, pollen provisions are the primary microbial inoculum available to solitary bee larvae and are hypothesized to be essential to support *Osmia* development (Dharampal *et al.*, 2020). However, while their exists significant relationships between the sources of pollen provisions and the bacterial composition of pollen provisions, supporting the view that brood cell microbial community derives largely from host-plant sources, the bacterial taxonomic identify across pollen sources is less predictable and locations effects which may be linked to diet breadth are paramount (Keller *et al.*, 2013; McFrederick and Rehan, 2019; Rothman *et al.*, 2020). Consequently, until we lean far more about solitary bee biology, we most carefully consider all sources of microbial inoculum in pollen provisions and assess the contributions and consequences of microbial diversity in bee development.

Supplemental References

Adler, L.S., Irwin, R.E., McArt, S.H., and Vannette, R.L. (2021) Floral traits affecting the transmission of beneficial and pathogenic pollinator-associated microbes. *Current Opinion in Insect Science* **44**: 1–7.

Álvarez, B., Biosca, E.G., and López, M.M. (2010) On the life of Ralstonia solanacearum, a destructive bacterial plant pathogen. *Current research, technology and education topics in applied microbiology and microbial biotechnology* **1**: 267–279.

Dharampal, P.S., Hetherington, M.C., and Steffan, S.A. (2020) Microbes make the meal: Oligolectic bees require microbes within their host pollen to thrive. *Ecological Entomology* **45**: 1418–1427.

Kapheim, K.M., Johnson, M.M., and Jolley, M. (2021) Composition and acquisition of the microbiome in solitary, ground-nesting alkali bees. *Scientific reports* **11**: 1–11.

Keller, A., Grimmer, G., and Steffan-Dewenter, I. (2013) Diverse microbiota identified in whole intact nest chambers of the red mason bee Osmia bicornis (Linnaeus 1758). *PLoS One* **8**: e78296.

Keller, A., McFrederick, Q.S., Dharampal, P., Steffan, S., Danforth, B.N., and Leonhardt, S.D. (2021) (More than) Hitchhikers through the network: The shared microbiome of bees and flowers. *Current Opinion in Insect Science* **44**: 8–15.

McFrederick, Q.S. and Rehan, S.M. (2019) Wild bee pollen usage and microbial communities co-vary across landscapes. *Microbial ecology* **77**: 513–522.

McFrederick, Q.S., Thomas, J.M., Neff, J.L., Vuong, H.Q., Russell, K.A., Hale, A.R., and Mueller, U.G. (2017) Flowers and wild megachilid bees share microbes. *Microbial ecology* **73**: 188–200.

Rothman, J.A., Cox-Foster, D.L., Andrikopoulos, C., and McFrederick, Q.S. (2020) Diet Breadth Affects Bacterial Identity but Not Diversity in the Pollen Provisions of Closely Related Polylectic and Oligolectic Bees. *Insects* **11**: 645.

Salanoubat, M., Genin, S., Artiguenave, F., Gouzy, J., Mangenot, S., Arlat, M., et al. (2002) Genome sequence of the plant pathogen Ralstonia solanacearum. *Nature* **415**: 497–502.

Salter, S.J., Cox, M.J., Turek, E.M., Calus, S.T., Cookson, W.O., and Moffatt, M.F. (2014) Reagent and laboratory contamination can critically impact sequence-based microbiome analyses. *BMC Biol* **12**:.

Vannette, R.L., McMunn, M.S., Hall, G.W., Mueller, T.G., Munkres, I., and Perry, D. (2021) Culturable bacteria are more common than fungi in floral nectar and are more easily dispersed by thrips, a ubiquitous flower visitor. *FEMS microbiology ecology* **97**: fiab150.

Voulgari-Kokota, A., Grimmer, G., Steffan-Dewenter, I., and Keller, A. (2019) Bacterial community structure and succession in nests of two megachilid bee genera. *FEMS microbiology ecology* **95**: fiy218.

Wicker, E., Lefeuvre, P., De Cambiaire, J.-C., Lemaire, C., Poussier, S., and Prior, P. (2012) Contrasting recombination patterns and demographic histories of the plant pathogen Ralstonia solanacearum inferred from MLSA. *The ISME journal* **6**: 961–974.

Supplemental Figure 1


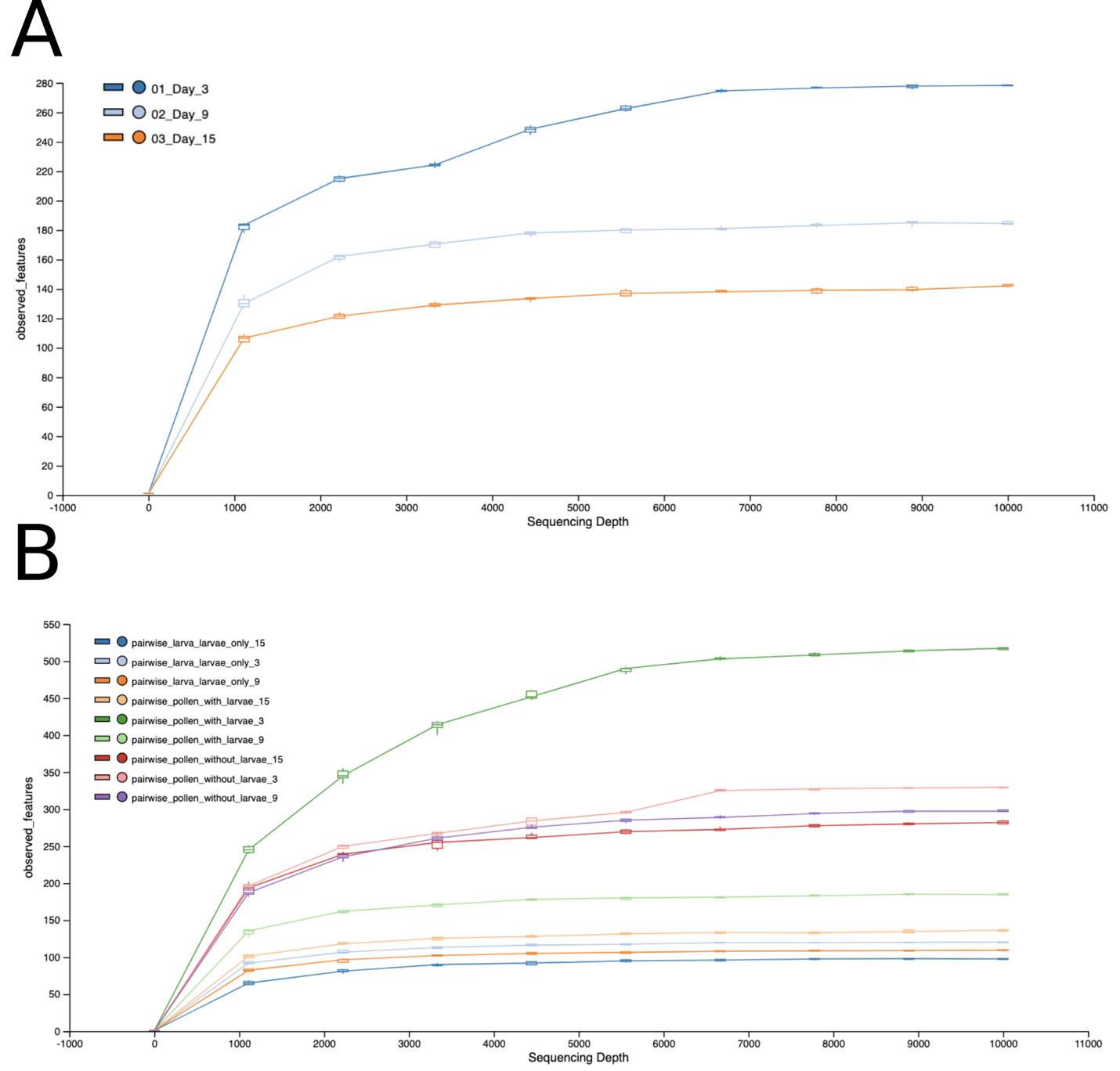


Supplemental Figure 1: Alpha rarefaction curves show ‘Observed features’ (ASVs) across sequencing depth (0-11,000). Figure (A) shows the rarefaction curves for all samples combined at day 3, 9 and 15. Figure (B) shows pollen only, pollen with larvae, and larvae only at each sampling day. Subsequently, a rarefaction depth of 4,690 was chosen to maximize the numbers of samples retained in our study while capturing most of the bacterial diversity present within the dataset.

Supplemental Figure 2


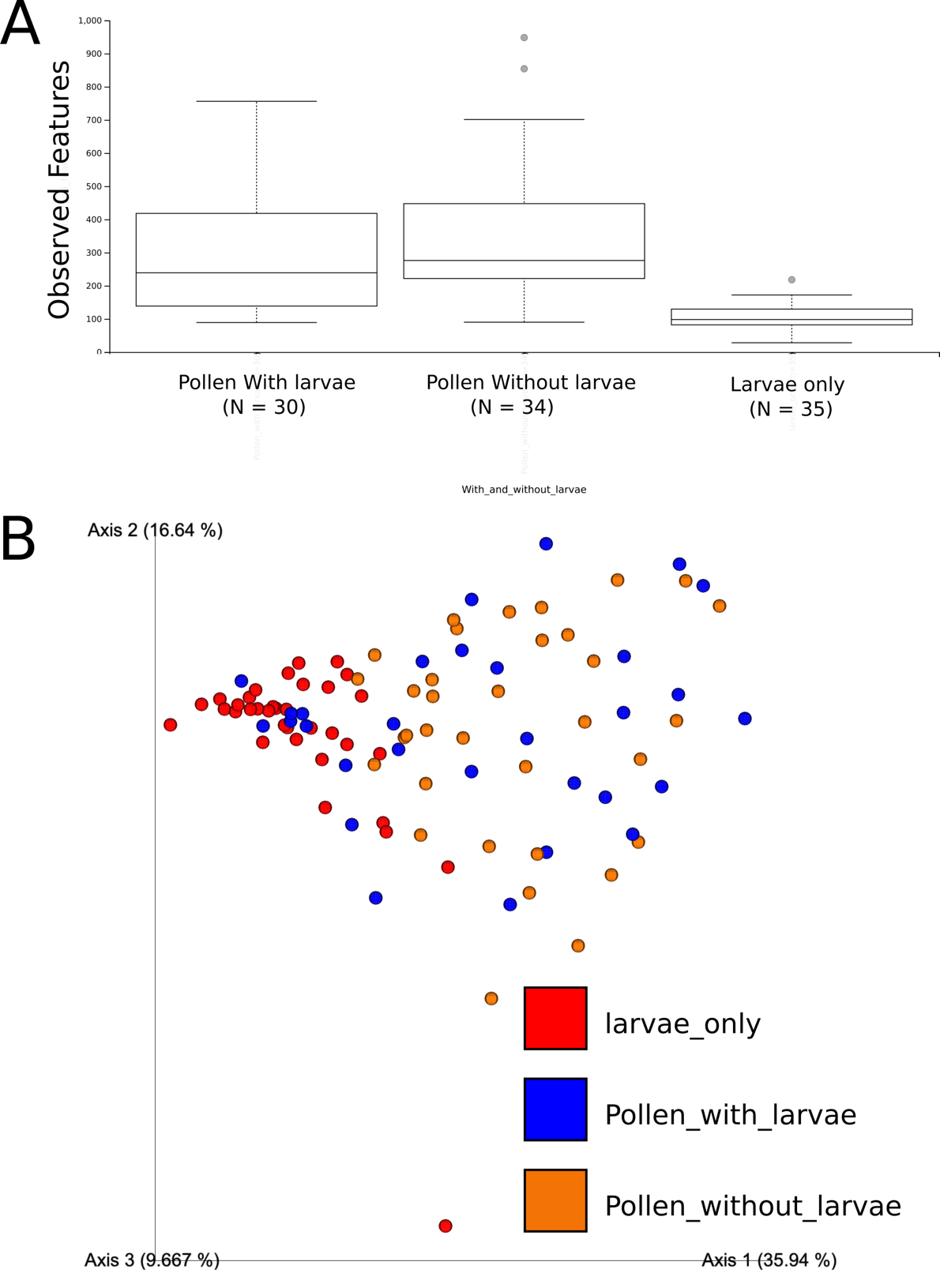


Supplemental figure 2) A comparisons of alpha (A) and beta diversity (B) of three sample types combined across three sampling days (day 3, day 9 and day 15).

Alpha diversity and beta diversity are significantly different amongst sample types (H = 54.479, p = 2.438 e-12), and (F = 10.93, p = 0.001), respectfully.

Supplemental Figure 3)


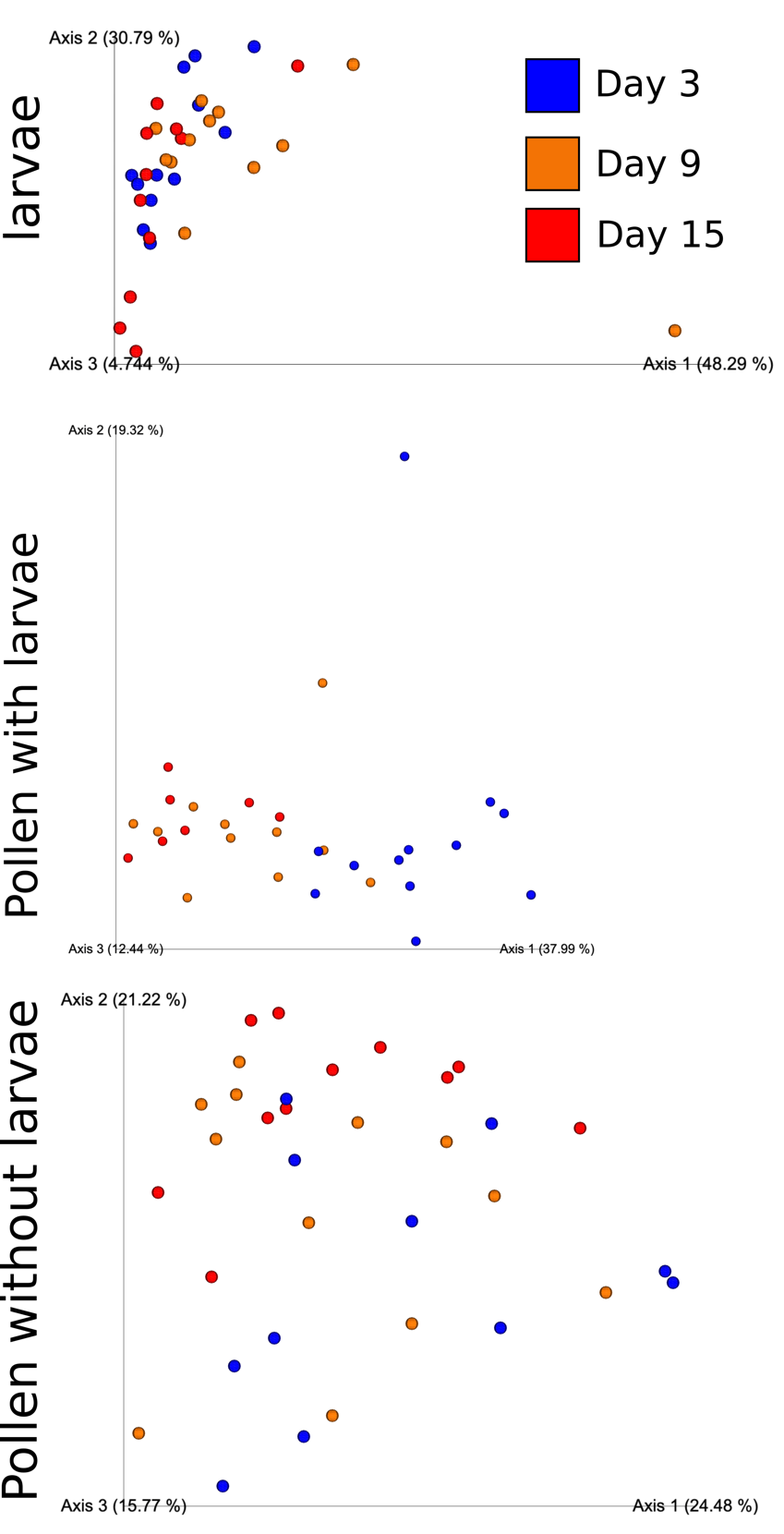


Supplemental Figure 3) A comparison of beta diversity for three sample types across three sampling days. Each circle represents a single sample and color indicates the sampling day. All three comparisons are significant with PERMANOVA: Larvae only (F = 2.42, p = 0.012), Pollen with larvae (F = 5.560, p = 0.001), and Pollen without larvae (F = 2.12, p = 0.006).

Supplemental Figure 4


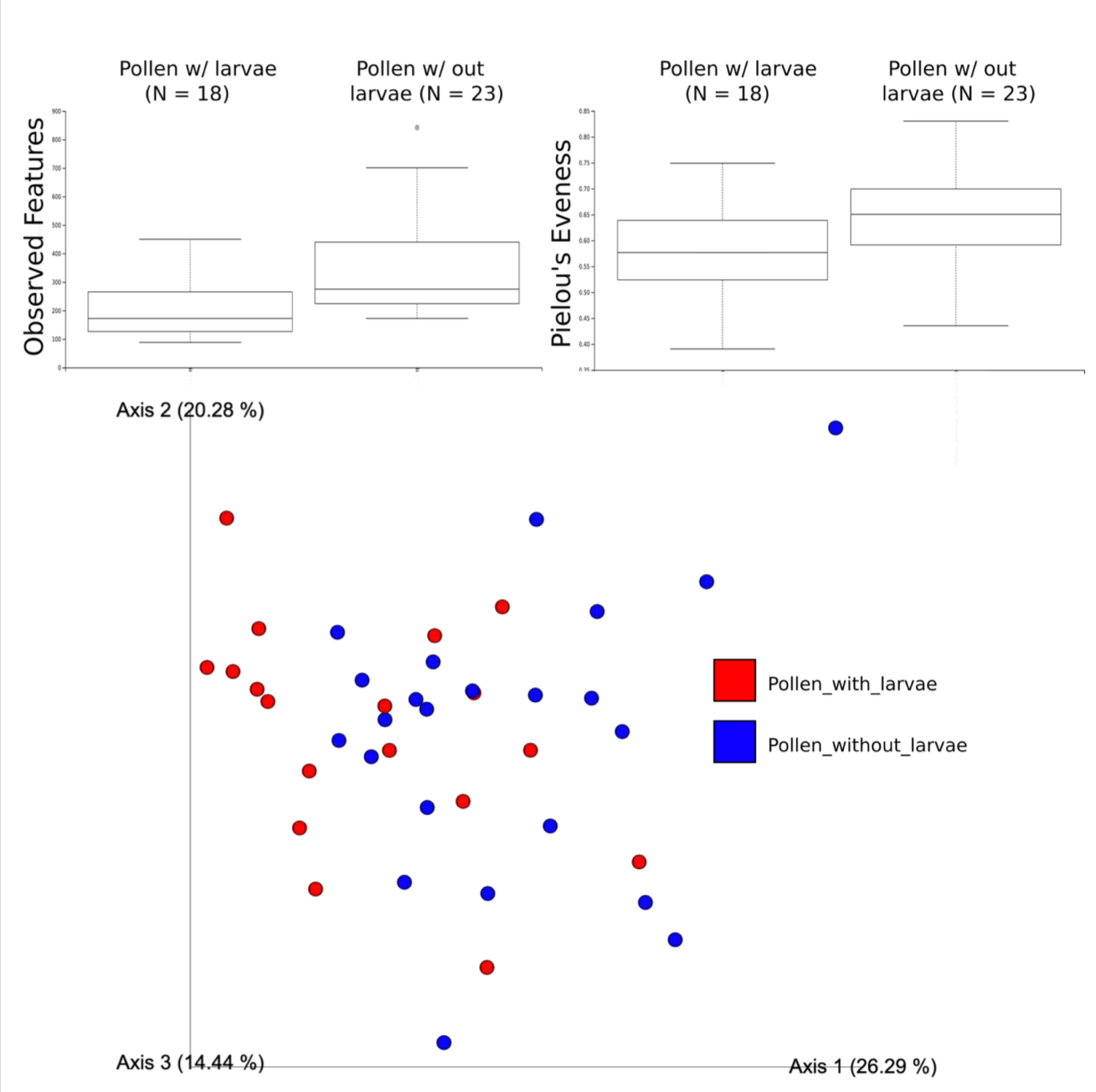


Supplemental Figure 4) Shows pollen with and without larva; day 9 and 15 are combined. We find differences in pollen-associated diversity occur by day 9 and the observation that larvae decrease the pollen microbial diversity is strengthened by combining these time points. Observed Features (H = 10.441, 0.001); Pielou’s Evenness (H = 6.901, p = 0.009); Beta diversity (F = 2.741, p = 0.008).

Supplemental Figure 5


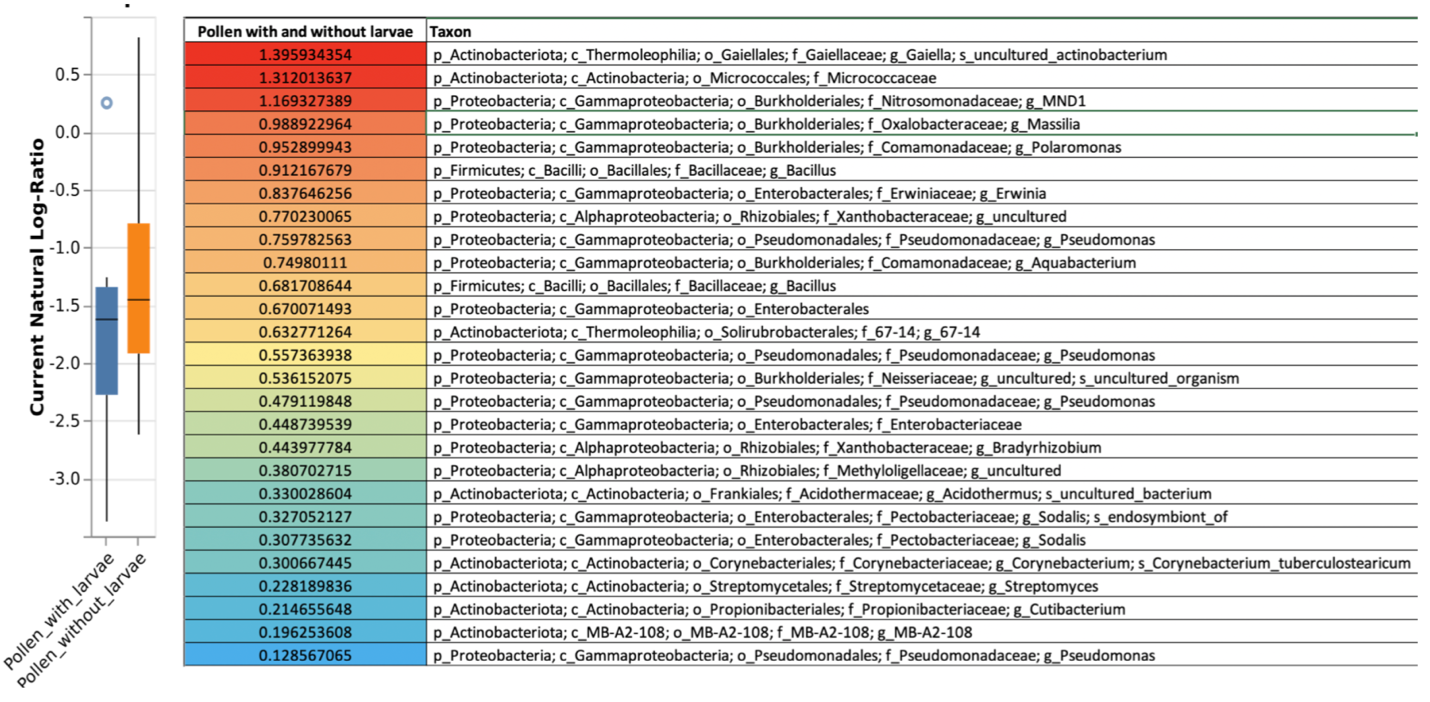


Supplemental Figure 5) Shows the bacterial features identified by Songbird whose presence contributes to differences detected between pollen with and without larval development. The data analyzed combines pairwise samples collected from day 9 and day 15.

Supplemental Figure 6


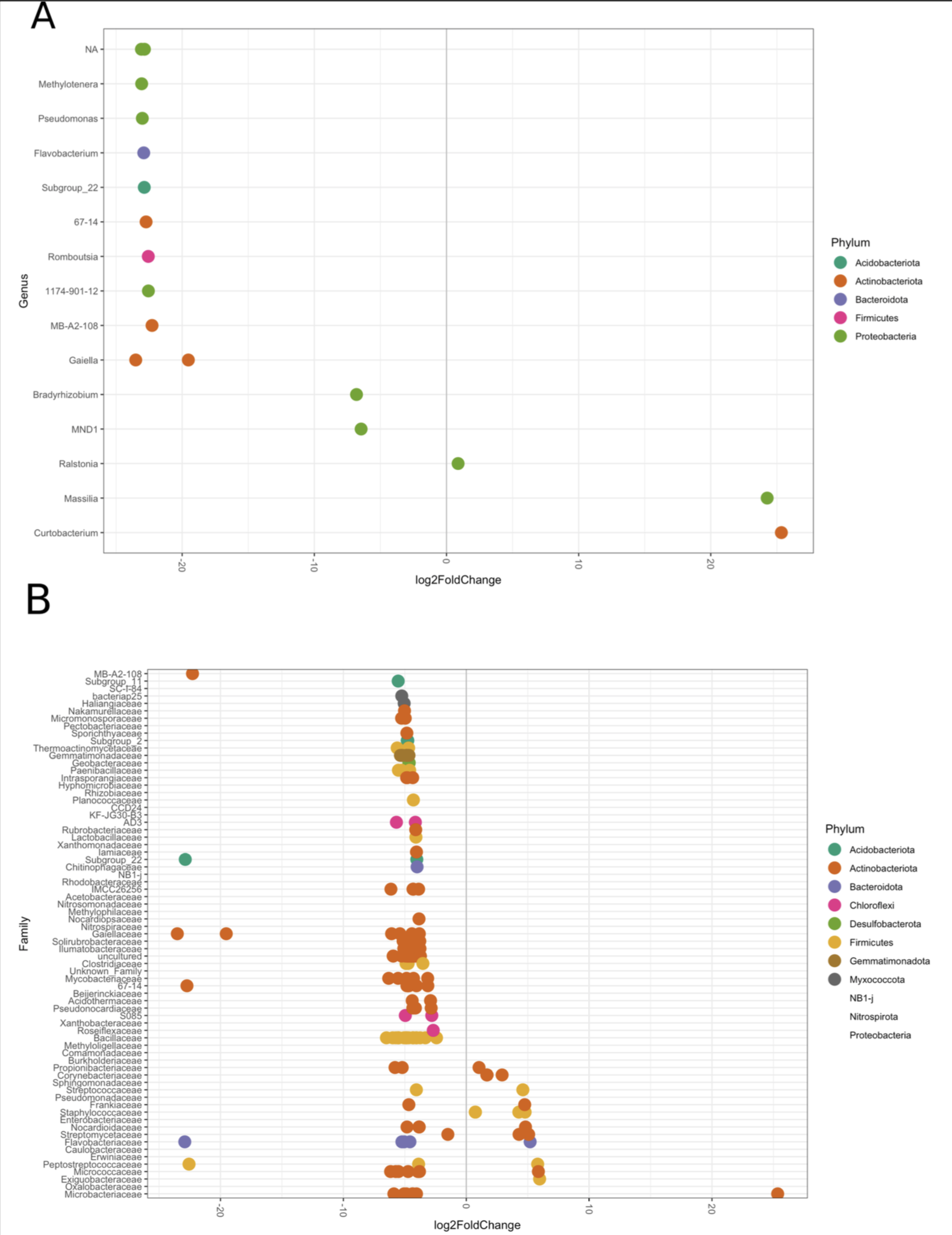


Supplemental figure 6) (A) Shows the taxa summarized the level of genus and color coded at the level of phylum that significantly differentiate (p< 0.05) between pollen with and without larvae that was combined by experimental day 9 and 15. A decrease in the log2 fold change reflects a bacterial taxon that has decreased abundance in pollen with larvae compared to pollen without larvae. (B) Shows the taxa summarized the level of family and color coded at the level of phylum that differ non-significantly (p = 0.5) between pollen with and without larvae that was combined by experimental day 9 and 15. The taxa that significantly differ (A) and the general pattern (B) convey a decrease in bacterial taxa associated with the presence of larvae at day 9 combine with day 15.

Supplemental Figure 7


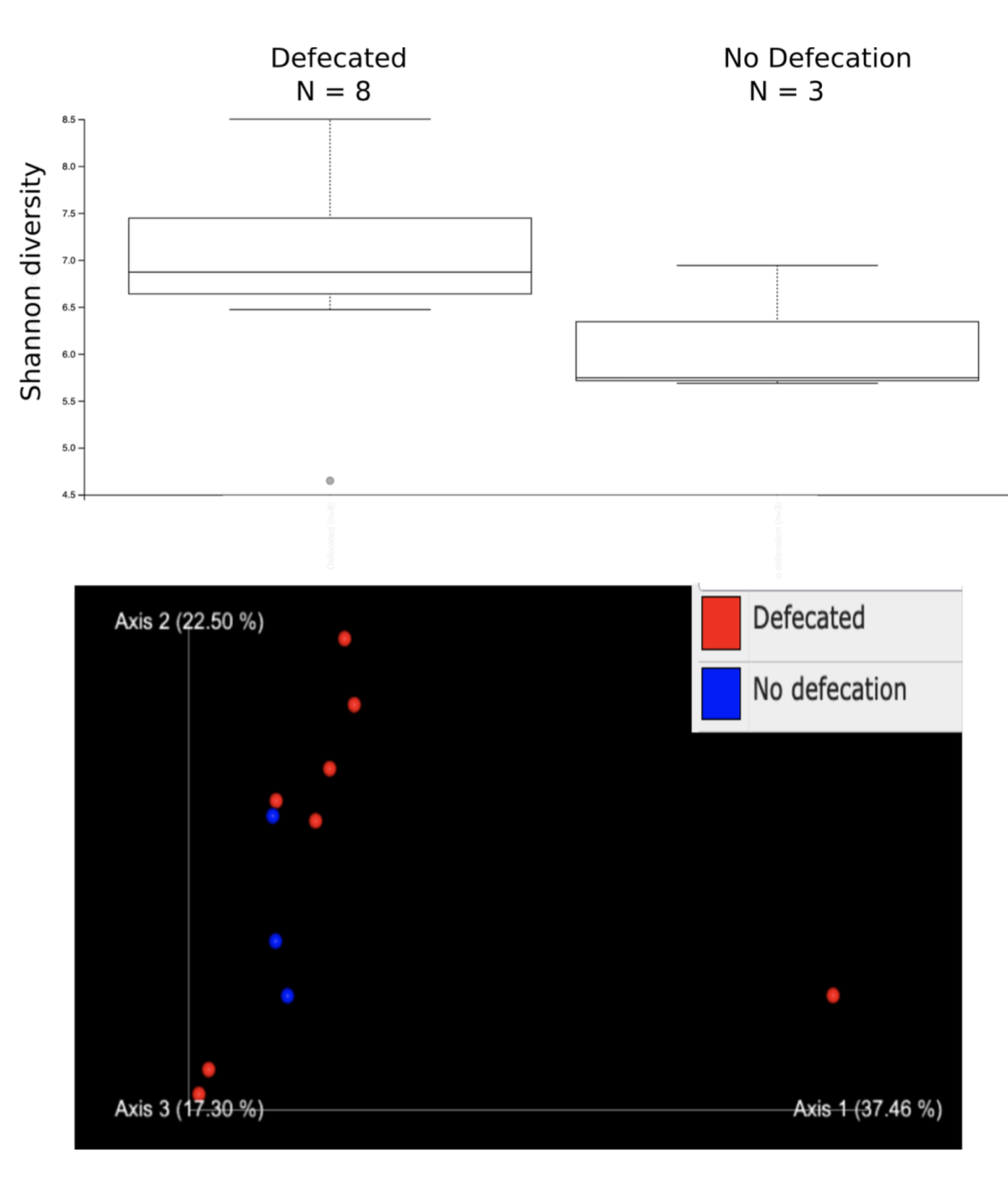


Supplemental Figure 7) Compares samples that had larvae that either defecated into the sample (red) or not defecated into the sample (blue). Shows non-significant differences in (A) and bacterial alpha diversity and (B) bacterial beta density for pollen samples collected at day 3.

Supplemental Table 1
